# Changes in sucrose metabolism patterns affect the early maturation of Cassava sexual tetraploid roots

**DOI:** 10.1186/s12870-022-03969-z

**Published:** 2022-12-10

**Authors:** Hanggui Lai, Yangjiao Zhou, Weiwen Chen, Yajie Deng, Yue Qiu, Xia Chen, Jianchun Guo

**Affiliations:** 1 Tropical Crops College of Hainan University, Haikou, 571104 China; 2 Hainan Forest Tree Seeds (Saplings) Terminal, Haikou, 570203 China; 3 College of Jiyang, Zhejiang A&F University, Zhuji, 311800 China; 4 Key Laboratory of Biology and Genetic Resources of Tropical Crops, Ministry of Agriculture, Institute of Tropical Bioscience and Biotechnology, Chinese Academy of Tropical Agricultural Sciences, Haikou, 571101 China

**Keywords:** Cassava, Sexual tetraploid, Storage roots, Sucrose metabolism, Early maturation, Regulatory mechanism

## Abstract

**Background:**

Cassava (*Manihot esculenta* Crantz) is an important multiuse crop grown for economic and energy purposes. Its vegetative organs are storage roots, in which the main storage material is starch. The accumulation characteristics of starch in cassava roots can directly affect the yield, starch content and maturation of cassava storage roots. In this study, we used a cassava sexual tetraploid (ST), which showed early maturation heterosis in previous work, as the main test material. We analyzed the sucrose metabolism and starch accumulation characteristics of the ST and its parents from the leaf “source” to the storage root “sink” during different developmental stages and explored the regulatory mechanisms of ST storage root early maturation by combining the transcriptome data of the storage roots during the expansion period.

**Results:**

The results showed that the trends in sucrose, glucose and fructose contents in the ST leaves were similar to those of the two parents during different stages of development, but the trends in the ST storage roots were significantly different from those of their parents, which showed high sucrose utilization rates during the early stage of development and decreased utilization capacity in the late developmental stage. Transcriptome data showed that the genes that were expressed differentially between ST and its parents were mainly involved in the degradation and utilization of sucrose in the storage roots, and four key enzyme genes were significantly upregulated (Invertase *MeNINV8/MeVINV3*, Sucrose synthase *MeSuSy2*, Hexokinase *MeHXK2*), while the expressions of key enzyme genes involved in starch synthesis were not significantly different.

**Conclusions:**

The results revealed that the pattern of sucrose degradation and utilization in the cassava ST was different from that of its parents and promoted early maturation in its tuberous roots. Starch accumulation in the ST from sucrose mainly occurred during the early expansion stage of the storage roots, and the starch content during this period was higher than that of both parents, mainly due to the regulation of invertase and hexokinase activities during sucrose metabolism. This study provides a basis for further genetic improvements to cassava traits and for breeding varieties that mature early and are adapted well to provide starch supply requirements.

## Background

Cassava (*Manihot esculenta* Crantz) originated in South America and is now commonly grown in many tropical and subtropical regions. The vegetative organs of cassava are storage roots, the main storage substance in the roots is starch, and cassava has the highest starch content among known starch crops. Due to its high degree of adaptability to its growing environment, cassava has become an important multiuse crop that is grown for economic and energy purposes [1, 2]. The main value of the cassava storage roots, apart from their edibility, is for starch processing [3]. With increasing demands for cassava starch and its subsequent processing products, yields of cassava cannot meet market demands, so it is crucial to select and breed excellent, high-yielding and early-maturing cassava varieties.

The accumulation characteristics of starch in cassava can directly affect the yield, starch content and maturation of cassava storage roots [4]. The raw material for starch synthesis in the roots is mainly derived from photosynthesis products in the source leaves, which are transported through the stem phloem to the roots and then degraded and utilized for starch synthesis [5]. It is generally believed that starch is synthesized from sucrose [6], and most cytoplasmic sucrose is catalytically degraded by sucrose synthase to decompose into fructose and uridine diphosphate glucose (UDPG), which is transformed into glucose-6-phosphate (Glu-6-P) and further forms glucose-1-phosphate (Glu-1-P). The key step of plant starch biosynthesis occurs in amyloplasts. ADP glucose pyrophosphorylase (AGPase) catalyzes the synthesis of ADP glucose (ADPG) from ATP and Glu-1-P with ADP glucose as the glucosyl donor for starch β-glucan synthesis. Finally, amylopectin is produced by soluble starch synthase (SSS), starch branching enzyme (SBE) and debranching enzyme (DBE), while the other part synthesizes amylose by granule-bound starch synthase (GBSS) [7–9]. In previous studies, we treated reproductive cells with a mixture of colchicine and dimethyl sulfoxide to obtain 2n male and female gametes and then successfully obtained a hybrid progeny cassava sexual tetraploid (ST) that showed a homozygous genotype and stable ploidy by introducing interspecific hybridization during the blooming period, and this hybrid showed a certain degree of heterosis relative to its parents [10]. During the process of studying the growth properties of ST during different developmental stages, we found that after sexual doubling, the ST not only changed its growth characteristics but also produced new variations, and its starch-filling period was reached early relative to that of its parents. Due to the filling of starch granules, the storage roots reached a turgid state approximately 5 months after planting during the expansion period, which was similar to the state of the cells observed during the mature period, but the starch-filling stage of its parents was between the late expansion and mature stages, with low levels of starch filling in the storage roots during the expansion period [10]. Correspondingly, the starch content of the ST also reached a high level at approximately 5 months after planting, and little starch was synthesized after this period. The starch content increased slightly, and the starch components also changed. Similarly, the yield of the ST storage roots was significantly higher than that of the parents during the expansion period, but the yield was nearly the same at harvest time (annual). The harvest time of the ST storage roots is earlier than that of its parents, which is an important manifestation of its new genetic improvements, but the regulatory mechanism by which early maturation relative to the parents is reached in the ST storage roots is not clear. Therefore, this study aims to compare and analyze the physiological, agronomic performance, sucrose metabolism and starch accumulation characteristics of the ST and those of its parents from the leaf “source” to the storage root “sink” during different periods of growth, and clarify the regulatory mechanisms leading to ST storage root early maturation by combining their transcriptome data (uploaded to SRA database of NCBI, login No: SRP151951) of the storage roots during the expansion period (165 days after planting), which provides a basis for further genetic improvements to cassava traits to breed excellent varieties that meet starch supply requirements.

## Results

### Comparison and analysis of basic agronomic traits of the cassava ST (ST) and its parents

**Fig. 1:**
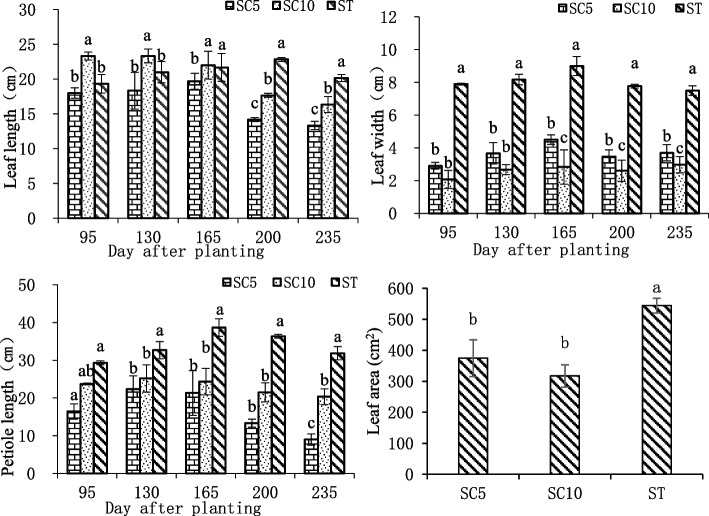
Comparison of changes in leaf length, decomposite leaf width, petiole length and leaf area of cassava ST and its parents during different growth stages. Different lowercase letters indicate significant differences at the 0.05 level (Duncan’s test)

The results of blade-related indices showed that the growth trend in ST leaf length was not consistent with that of the parents. The leaf length of SC5 and SC10 reached their maxima at 130 days after planting, while that of ST reached a maximum at 200 days after planting and then continued to show values significantly higher than those of the parents. The decomposition leaf width of the ST was significantly longer than that of both parents during each period of growth, and the petiole length of the ST was significantly longer than that of both parents 95 days after planting. The difference in leaf area between the ST and SC5/SC10 reached a significant level, with the leaf area of the ST being approximately 50.81% higher than that of the diploid parent SC5 and approximately 72.59% higher than that of the diploid parent SC10, and the difference in leaf areas between the two parents was not significant (Fig. 1), indicating that the cassava ST had increased leaf area and exhibited distinct polyploid giantism characteristics.

**Fig. 2:**
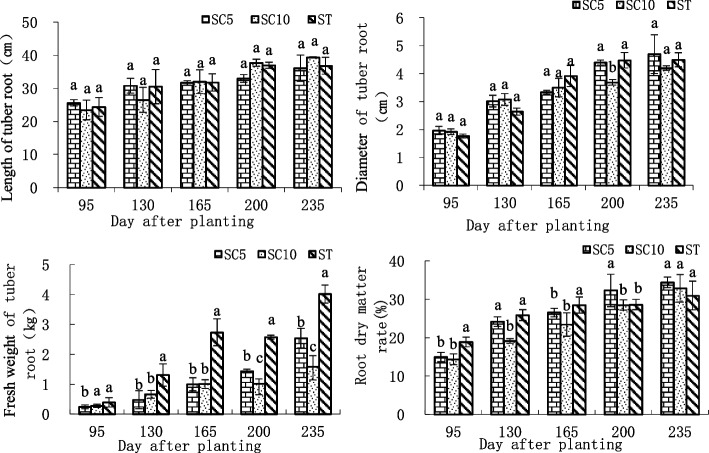
Changes in storage root length, diameter, fresh weight and dry matter rate of the cassava ST and its parents during different growth stages. Different lowercase letters indicate significant differences at the 0.05 level (Duncan’s test)

The results of storage root-related indices showed that the storage root length of ST and its parents SC5/SC10 showed similar trends, with continuous elongation during development and no significant difference in each period. The root diameter of the three varieties showed the same trends, that is, they increased with development, and the diameter of the ST storage roots was larger than that of the parents 165 days after planting, which was not significantly different from that of SC5 and was significantly different from that of SC10. The average fresh weight of the roots of the three varieties presented similar trends. They increasing with growth, but the average fresh weight of the ST roots was significantly greater than that of the parents after the expansion period. The trend in the storage root dry matter rate of the ST was not consistent with that of the parents. The SC5/SC10 dry matter rate increased with development, while the ST dry matter rate peaked at 165 days after planting and was slightly higher than that of both parents, rising slowly thereafter. Later in development, it was slightly lower than that of the parents, but the difference was not significant (Fig. 2). The above results showed that there were no variations in the storage root length of the ST, while the ST had a slight advantage in terms of its storage root diameter traits, showed heterosis in storage root yield and dry matter accumulation during the expansion period and had a shortened maturation period.

### Analysis of photosynthetic characteristics and chlorophyll content in the leaves of the ST and its parents

An increase in leaf area can affect the photosynthesis of plant leaves. Photosynthesis is an important metabolic activity in plant leaves; it converts carbon dioxide and water into organic matter, and its strength is of great significance to cassava development, storage root yield and plant stress resistance. The photosynthetic characteristics of the cassava ST and its parents were measured, and it was found that the net photosynthetic rate, transpiration rate, stomatal conductivity and water use efficiency of the ST were significantly different from those of the two parents SC5/SC10. The net photosynthetic rate and water use efficiency of the ST were 22.51 and 5.74% higher than those of SC5 and 33.67 and 20.11% higher than those of SC10, respectively. The intercellular CO_2_ concentration of ST was lower than that of the parent SC5, but the difference was not significant (Table 1).

**Table 1:** Photosynthetic characteristics of cassava sexual tetraploids and their parents

Parameters	SC5	SC10	ST
Net photosynthetic rate (μM·m^−2^·s^− 1^)	8.47 ± 0.68 b	7.25 ± 1.88 b	10.93 ± 1.35 a
Transpiration rate (mM·m^− 2^·s^− 1^)	4.08 ± 0.74 ab	3.94 ± 0.66 b	5.37 ± 0.57 a
Stomatal conductivity (M·m^− 2^·s^− 1^)	0.25 ± 0.06 b	0.16 ± 0.04 b	0.36 ± 0.04 a
Intercellular CO_2_ concentration (μL·L^− 1^)	172.52 ± 5.22 a	168.69 ± 12.11 a	170.30 ± 4.52 a
Water use efficiency (μM·mol^− 1^)	2.09 ± 0.55 b	1.84 ± 0.67 c	2.21 ± 0.43 a

Different lowercase letters indicate significant differences at the 0.05 level (Duncan’s test)

Chlorophyll is the most important pigment in plant photosynthesis and plays a central role in light absorption; therefore, it has a direct effect on the photosynthetic rate of plant leaves and subsequently affects photosynthetic products. The chlorophyll content of the cassava ST and its parents was determined, and we found that the total chlorophyll, chlorophyll a and chlorophyll b contents of the ST were significantly higher than those of the two parents, followed by those of SC5 and SC10 (Table 2), which coincided with the photosynthetic parameters of the three cassava cultivars (Table 1).

**Table 2:** Chlorophyll content of cassava STs and their parents

Variety	Chlorophyll a (mg/g FW)	Chlorophyll b (mg/g FW)	Total chlorophyll (mg/g FW)
SC5	1.947 ± 0.042 b	0.879 ± 0.088 b	2.889 ± 0.052 b
SC10	1.834 ± 0.131 b	0.859 ± 0.025 b	2.752 ± 0.056 c
ST	2.113 ± 0.119 a	0.952 ± 0.011 a	3.133 ± 0.022 a

Different lowercase letters indicate significant differences at the 0.05 level (Duncan’s test)

### Analysis of starch content in storage roots of the ST and its parents

**Fig. 3:**
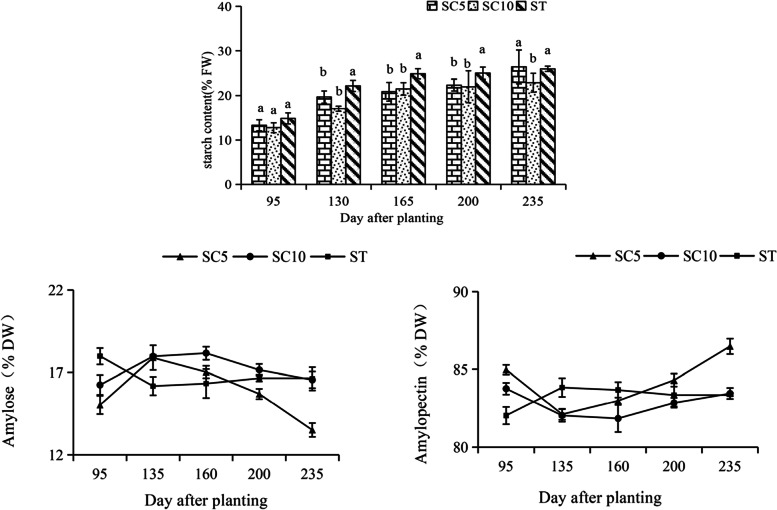
Comparison of total starch, amylose and amylopectin contents in the storage roots of the cassava ST and its parents during different developmental stages. Different lowercase letters indicate significant differences at the 0.05 level (Duncan’s test)

The storage root starch content of the three cassava varieties was measured at different times. The results showed that the starch content of the ST increased significantly from 95 to 165 days after planting and was significantly higher than that of the parents during the expansion period, while the trend in increasing starch content slowed after 165 days, and the starch content did not increase significantly. However, the starch content of the two parents showed a gradual increase during development. In addition, the trend in the ST starch content (amylose/amylopectin content) was opposite to that of its parents. The amylopectin content of the ST showed an increasing tendency and then decreased during storage root development, while that of both parents decreased and then increased. During the expansion period, the ST had a higher amylopectin content, but there was no significant difference between that of its parents (Fig. 3). The results revealed that the starch-filling period of the cassava ST was different, showing an earlier period than that of the parents, and the straight/branched chain starch ratio was also different.

### Analysis of the soluble sugar content in the functional leaves, stem phloem sap and storage roots of the ST and its parents

**Fig. 4:**
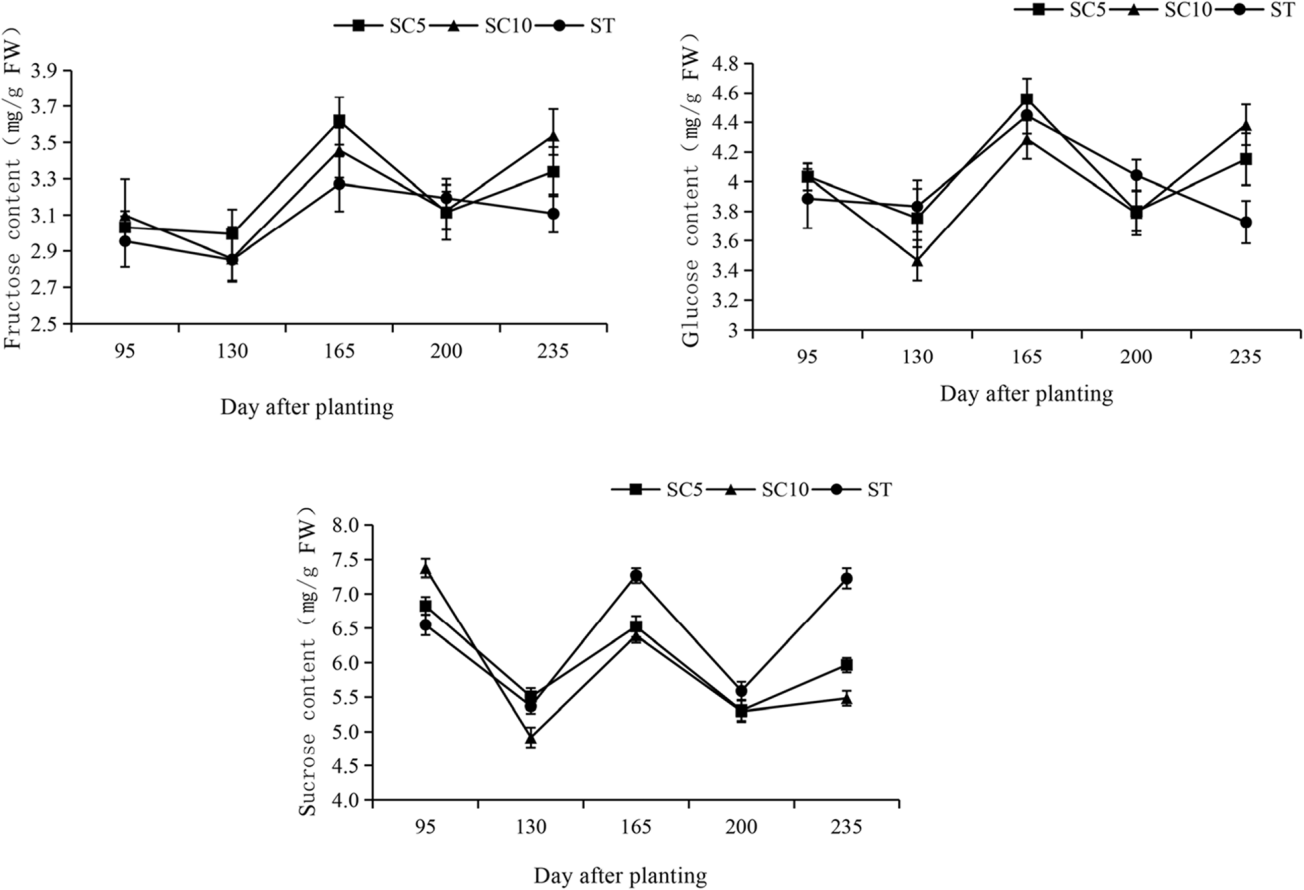
Soluble sugar content in the leaves of cassava ST and its parents

**Fig. 5:**
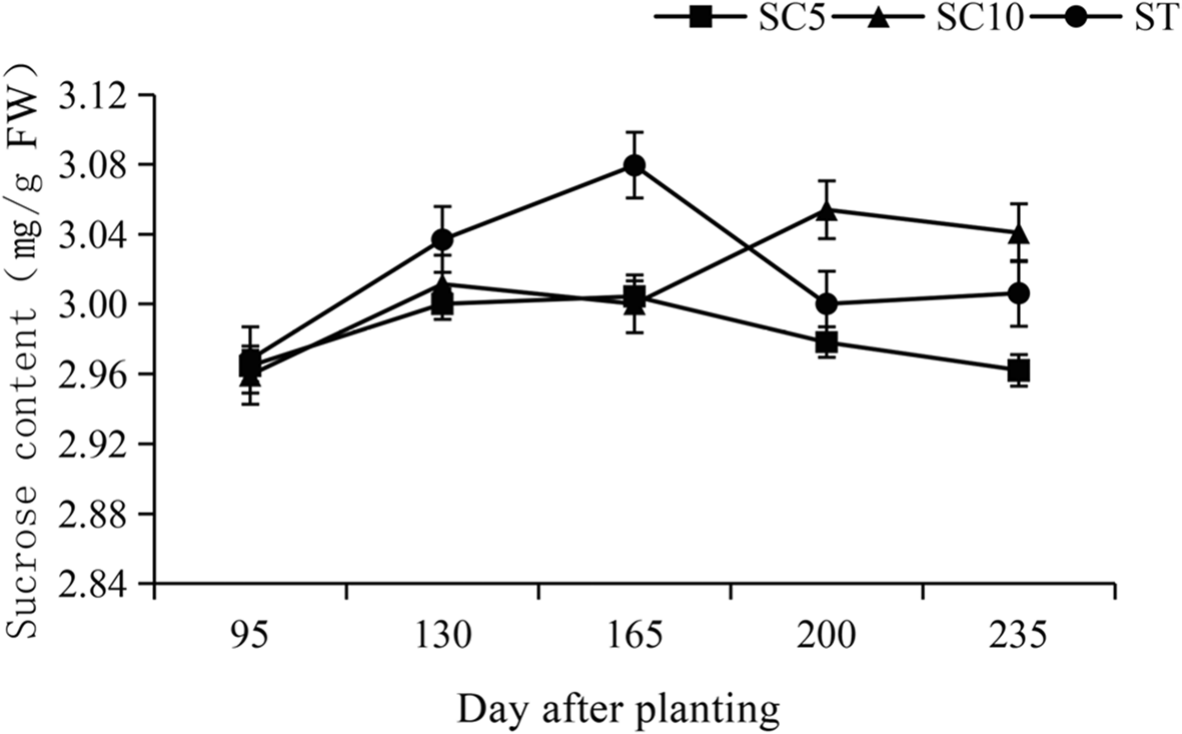
Soluble sugar content in the stem phloem sap of the cassava ST and its parents

**Fig. 6:**
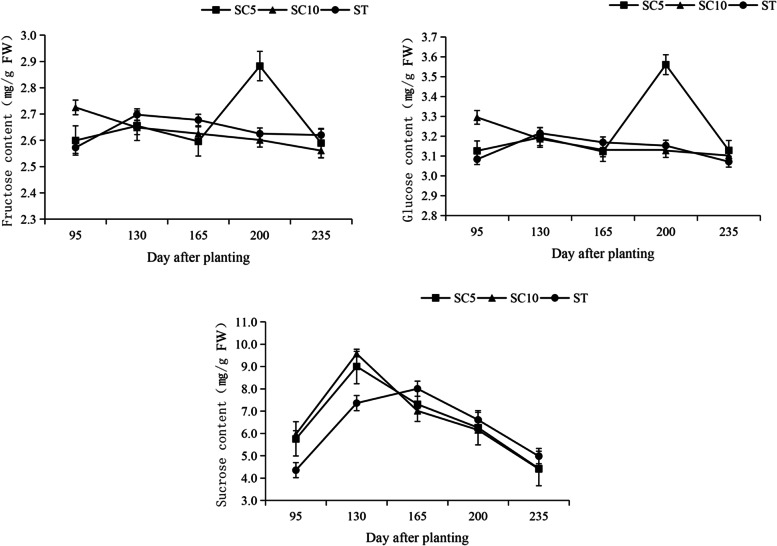
Soluble sugar content in the roots of the cassava ST and its parents

Sucrose is the raw material for starch synthesis, and its synthesis in leaves (source organ), transport in stem phloem and speed of metabolism in storage roots (sink organ) could affect starch formation and the yield of the harvested organ. The soluble sugar content of the functional leaves, stem phloem sap and storage roots of the cassava STs and their parents were measured during different growth periods, and it was found that the trends in the sucrose, glucose and fructose contents in the ST leaves during the developmental period were similar to those of the parents (Fig. 4). In cassava stems, the raw material was mainly transported to the storage roots in the form of sucrose, so here, only sucrose was detected in the stem, while fructose and glucose were barely detected. The sucrose content in the ST stems was significantly higher than that of the two parents during the early stage of development and decreased during the later stage of development (Fig. 5). The utilization trend for the soluble sugar in the ST storage roots during different developmental stages was not consistent (Fig. 6).

At approximately 130-150 days of development, the three cassava varieties did not differ significantly with respect to the amount of sucrose synthesis in the source leaves, but in the stems, the ST transported significantly more sucrose than its parents during this period, while it stored significantly less sucrose in its roots and more fructose and glucose than its parents, suggesting that the ST had a high rate of sucrose catabolism and that starch synthesis probably began to occur rapidly during this period, while the parents had weak capacities to utilize sucrose during this period.

At approximately 165 days of development, the ability of the three cassava varieties to synthesize sucrose in the source leaves increased significantly, and the ST had a significantly higher sucrose content than its parents, which was related to the large leaf area and strong photosynthetic characteristics of ST. The sucrose transport capacity of the ST stems continuously improved during this period. Sucrose began to accumulate in the ST storage roots and increased at this time, while the sucrose in both parents began to be utilized, resulting in a gradual decrease.

After 165 days of development, the ability of the ST to synthesize sucrose from source leaves was significantly stronger than that of both parents, but the sucrose transport capacity of its stem began to decrease, which was probably due to the accumulation of sucrose in the storage roots and the slower consumption of sucrose. During this period, the sucrose utilization rate of the ST was significantly lower than that of the two parents, while its parents showed a trend toward rapid sucrose degradation, with SC5 showing a high increase in fructose and glucose content in the storage roots at 200 days of development. This result suggested that the reduced sucrose utilization capacity of the ST storage roots during the later stage of growth is the reason why its starch content was lower than that of the parents.

### Screening key enzyme genes for starch and sucrose metabolism differentially expressed in the storage roots of the ST and its parents

To understand the molecular mechanism of early maturation in the ST storage roots, this study combined the transcriptome of the cassava ST storage roots with that of its parents during the expansion period (165 days after planting) to screen differentially expressed genes of key enzymes involved in the starch synthesis metabolic pathway. The transcriptome data showed that in starch and sucrose metabolism, 35 differentially expressed genes in the ST accounted for 6.61% of the total differentially expressed genes relative to SC5, and 40 differentially expressed genes in ST accounted for 6.77% of the total differentially expressed genes relative to SC10.

**Fig. 7:**
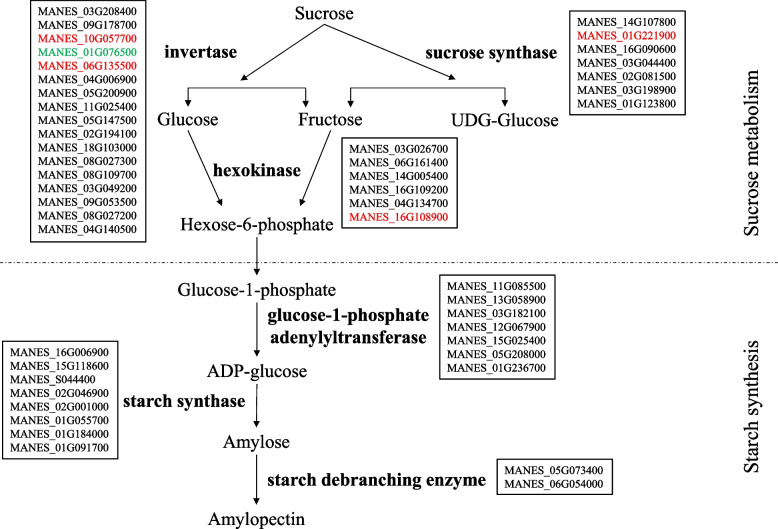
Key enzyme genes involved in starch synthesis. Red represents the upregulated expression of genes; green represents the downregulated expression of genes

To improve the pertinence of gene screening, we further screened differentially expressed genes under the conditions of FDR < 0.05 and | log2fc | > 2. Starch synthesis in the storage roots could be divided into 2 steps: sucrose metabolism and starch synthesis. The screening results showed that the differentially expressed genes in the storage roots of ST and its parents during the expansion period were mainly involved in sucrose decomposition and utilization; these genes included *MeNINV8* (MANES_10G057700), *MeVINV3* (MANES_06G135500), *MeSuSy2* (MANES_01G221900) and *MeHXK2* (MANES_16G108900), which were significantly upregulated and included *MeVINV2* (MANES_01G076500), which was downregulated. The log2FC value of the differentially expressed *MeHXK2* in FPKM reached 13.14, and this gene was barely expressed in SC5 but was highly expressed in the ST. In addition, there was no significant difference in the expression of key enzyme genes during the starch synthesis phase under the screening condition (Fig. 7). The results showed that starch and sucrose metabolism were altered in the ST storage roots relative to the parents during the expansion period, which affected starch synthesis, further demonstrating that the starch content of the ST roots during the expansion phase was higher than that of the parents, mainly through the regulation of sucrose metabolism.

### Analysis of key enzyme activities in sucrose and starch metabolism

**Fig. 8:**
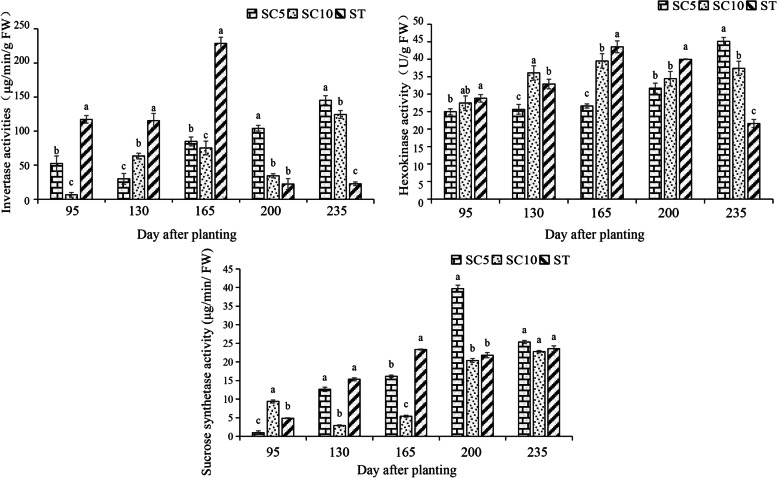
Comparison of the invertase, sucrose synthase and hexokinase activities of the cassava ST with that of its parents during different stages of growth

The activities of the key enzymes (invertase, sucrose synthase, hexokinase) associated with the differentially expressed genes were measured in the ST and its parents during different stages of development. The results showed that invertase activity was significantly higher in the ST than in the parents during the period of storage root expansion (95-165 days), especially at 165 days after planting, indicating that invertase was active during this period and could degrade the sucrose transported from the source leaves to the storage roots and use it as a raw material for starch synthesis. The activity of the enzyme was greatly attenuated and remained at a low level during the later growth period (200-235 days), whereas the parents showed a trend toward increasing enzyme activity (Fig. 8), indicating that the degradation and utilization of sucrose by invertase was a key factor in the synthesis of ST starch in the early stage of development and in the decrease in sucrose content during the later growth stage, which eventually led to early maturation in the storage roots.

The sucrose synthase activity of the ST storage roots was higher than that of the SC10 roots 130 days after planting but not significantly different from that of the SC5 roots. At 165 days after planting, its activity was significantly higher in the ST. During the later stage of development, the sucrose synthase activity of SC5/SC10 increased significantly, and the enzyme activity of ST remained at a higher level that was not significantly different from that of the parents (Fig. 8). The results showed that sucrose synthase and invertase were synergistically involved in sucrose catabolism and starch synthesis in the storage roots during the ST expansion period, promoting the early filling of storage root starch, but they were not key enzymes in the reduction in starch content in the ST roots during the later developmental stage.

The hexokinase activity of the ST storage roots was consistently and significantly higher than that of SC5 during the expansion period (95-200 days), but there was no distinct regularity compared with SC10. At 235 days after planting, the ST hexokinase activity decreased significantly, and the enzyme activity was significantly lower than that of the parents (Fig. 8). Hexokinase is not only a key enzyme involved in the early maturation of ST storage roots during the expansion period but also a key enzyme that affects starch content, which no longer increased in the later stage of root development and resulted in a decrease in the dry matter rate.

## Discussion

### Effect of sexual polyploidization on the growth characteristics of cassava

Plant polyploids tend to have significantly larger tissues, organs, cells and nuclei, mainly in the roots, stems, leaves, fruits, flowers and seeds, due to the dose effect of genome replication [11–13]. Therefore, plant polyploidy is usually accompanied by the following growth characteristic: strong plants, strong growth potential, developed roots, thick leaves, increased leaf type and leaf area, reduced number of leaves, reduced number of branches and increased fruit organs [14–16]. Bai [17] found that polyploid plants were significantly larger than diploid controls in phenotypes such as plant height, crown size and stem diameter when the polyploid *Salvia miltiorrhiza* was bred for cultivar improvement. The leaf morphology of *Betula platyphylla* tetraploids showed significant gigantism, with leaf areas and stomatal sizes exceeding those of diploid controls by more than 10 and 90%, respectively, and its reproductive characteristics also showed gigantism, with male and female flowers, fruit infructescence and pollen being significantly larger than those of diploid controls [18]. In addition, naturally mutated cassava polyploids [19] and artificial cassava asexual polyploids [20] were also found to have gigantic phenotypic characteristics. Here, the growth characteristics of ST cassava were studied, and it was found that cassava STs also exhibited gigantic growth characteristics relative to their parents, with their leaves showing significant gigantism. The leaf area was approximately 50.81% larger than that of the diploid parent SC5 and 72.59% larger than that of the diploid parent SC10, and the petiole length was also much longer than that of its parents (Fig. 1). The ST harvested storage root organs were also significantly larger than those of the diploid parents during the expansion period (Fig. 2). The analysis showed that the growth characteristics of the cassava STs were consistent with those of general polyploid plants, which have substantial potential for the genetic improvement and breeding of cassava.

### Sexual polyploidy improves the photosynthetic characteristics and chlorophyll content of cassava leaves

Photosynthesis is the source of plant energy and an important metabolic activity of leaves. In cassava, leaves provide raw materials for sink organ development through photosynthesis, the strength of which directly affects the development and storage root yield of cassava [21]. A large number of studies have shown that changes in plant ploidy have a substantial impact on the photosynthetic rate of leaves. Chen et al. [22] studied the photosynthetic characteristics of heteropolyploid melons with different ploidies and found that the leaf photosynthetic rates increased with increasing ploidy. The net photosynthetic rate of tetraploid *Echinacea purpurea* leaves have also been shown to be significantly higher than that of diploid leaves during development [23]. The leaf photosynthetic rate of the cassava South China No. 8 autotetraploid inducer was also higher than that of diploid plants [24], which is consistent with our experimental results. The net photosynthetic rate, transpiration rate, stomatal conductivity and water use efficiency of the cassava ST reached significant levels relative to both parents, with a net photosynthetic rate that was 22.51% higher than that of SC5 and 33.67% higher than that of SC10 (Table 1). The net photosynthetic rate directly reflects the ability of cassava leaves to synthesize raw materials, and a higher transpiration rate indicates greater physiological metabolic activity, suggesting that cassava STs have stronger photosynthetic abilities than their diploid parents, which is one of the reasons for their increased biomass and vigorous plant growth.

Compared with the diploid parents, the contents of total chlorophyll, chlorophyll a and chlorophyll b in the leaves of the cassava STs were significantly higher (Table 2), which is consistent with the results of previous studies [25]. Cao et al. [26] suggested that the efficiency of light energy capture by PSII reaction centers was higher when plants had more chlorophyll within leaves, which led to an enhancement in the photosynthetic rate. An et al. [24] showed that the increased photosynthetic rate in cassava autotetraploid leaves was due to the strong light harvesting capacity and high efficiency of photochemical transformation in leaf PS II reaction centers. Chlorophyll is the most important pigment in plant photosynthesis and plays a central role in light absorption during photosynthesis; therefore, the increased chlorophyll content of cassava ST is an important basis for its improved photosynthesis, but its relationship with the efficiency of light energy capture by the PSII reaction center needs to be further studied. In addition, Lu et al. [27] suggested that the middle decomposite leaf length of cassava was positively correlated with photosynthesis in leaves. In this study, the middle decomposite leaf length of the cassava ST was significantly higher than that of both parents, which may also be related to its enhanced photosynthetic rate.

### Altered patterns of sucrose metabolism in the roots/stems/leaves of the cassava sexual polyploids regulate the early maturation of storage roots

The assimilates of photosynthesis in the source leaves of cassava are usually transported in the form of sucrose [28], which is transported to the sink organ through the stem phloem as the raw material for starch synthesis [7, 29], while the ability of sink cells in the sink organ to metabolize and utilize sucrose and synthesize starch is critical to the yield of cassava storage roots [30, 31]. Previous studies have clarified that the sucrose content in cassava leaves is positively correlated with the starch content in the storage roots, the sucrose content in stems is positively correlated with starch accumulation in the roots, and in roots, the sucrose content is significantly and negatively correlated with starch accumulation [21, 27, 32]. In this study, we observed the dynamic accumulation of sucrose in the leaves of the cassava ST and its parents during different periods of growth and found that the accumulation of sucrose in the leaves of the cassava ST was slightly lower than that of the two parents during the storage root formation period, the sucrose content in the stem phloem was not significantly different from that of the two parents, and the accumulation of sucrose in the storage roots was also lower than that of its parents. At this time, there was no significant difference in the content of starch in the storage roots of the ST and its parents. During the storage root expansion period, the sucrose accumulation in the leaves and stems of the cassava ST was significantly higher than that of the parents, while sucrose accumulation in its storage roots was lower than that of the parents. The starch content of the ST roots was higher than that of their parents at this time, and the difference was significant. During the mature period, the trend in sucrose accumulation in the cassava ST leaves was more noticeable than that of the parents, but the sucrose content in its stems decreased because sucrose began to accumulate in the storage roots during this period at levels that exceeded that of its parents, which limited the transport capacity of the stem phloem. At this point the starch content of the roots was still higher than that of the two parents but was no longer increasing. The results revealed that the cassava ST source leaves had stronger sucrose synthesis abilities than that of the parents, which was related to their high photosynthetic efficiency. The sucrose loading and transport ability of the ST stem phloem was also significantly improved, which may be related to the fact that the polyploid stem was significantly thicker than that of the parents. However, the utilization of sucrose in the ST storage roots was mainly concentrated in the formation period and expansion period, with a reduced capacity for sucrose degradation and utilization and a slower trend toward increasing starch content in the later stages of development. These results were also found to be consistent with starch content via measurements of the fresh weight yield of the cassava ST storage roots (Figs. 3, 4, 5 and 6).

The relationship between sucrose accumulation, starch content and fresh weight of the storage roots in the cassava ST indicated that its storage roots could mature within a shorter period than those of its parents, showing early maturation hybrid superiority. Moreover, in our previous study [10], in which we observed the starch grains of the cassava ST and its parents by transmission electron microscopy, we found that the ST storage roots were fully filled with starch granules during the expansion period and did not significantly differ from those during the mature period, whereas the starch granules in the two parents did not being to fill during the expansion period; this provides additional evidence for the early maturation of the cassava ST storage roots.

In summary, the early maturation of the cassava ST storage roots is associated with changes in sucrose utilization patterns. The degradation and utilization of sucrose in cassava is regulated by a series of enzymes related to sucrose and starch metabolism during its growth. Sucrose synthesized in source leaves must be degraded into monosaccharides before they are transported to storage roots for starch synthesis [33]; thus, starch synthesis in cassava storage roots can be divided into two stages: sucrose degradation and starch synthesis. The main enzymes involved in sucrose catabolism are invertase (EC 3.2.1.26), sucrose synthase (EC 2.4.1.13), and hexokinase (EC 2.7.1.1), and the main enzymes involved in starch synthesis are ADP glucose pyrophosphorylase (AGPase, EC 2.7.7.27), starch synthase (SS, EC 2.4.1.21), and starch branching enzyme (SBE, EC 3.2.1.68). Invertase can catalyze β-1,2 glycosidic bonds and degrade sucrose into glucose and fructose, which participate in the subsequent metabolism, and sucrose synthase can convert glucose from sucrose to UDP, catalyzing the formation of UDPG and fructose to further participate in metabolism. This process is reversible. The catalytically formed product is phosphorylated by hexokinase to form glucose-6-phosphate (Glu-6-P), which is transported via the transporter to amyloplasts for starch synthesis [34]. Sucrose catabolic enzymes clearly play a key role in starch synthesis [35]. After Glu-6-P is transformed into glucose-1-phosphate (Glu-1-P), ADPG and PPi are catalyzed by ADP glucose pyrophosphorylase to form amylose under the action of starch synthase or amylopectin catalyzed by branching enzymes [7, 36]. In this study, we combined transcriptome data and found that enzyme genes related to sucrose degradation and utilization were differentially expressed in ST and its parents during starch accumulation during the storage root expansion period, including one hexokinase gene that was significantly upregulated (*MeHXK2*), one sucrose synthase gene that was significantly upregulated (*MeSuSy2*) and two invertase genes that were significantly upregulated, which included one vacuolar invertase gene (*MeVINV3*) and one neutral/alkaline invertase gene (*MeNINV8*). *HXK2*, which was highly significantly and differentially expressed compared with the parent SC5, has been shown to be a key hexokinase gene for hexose phosphorylation during cassava storage root development and to play an important role in sucrose metabolism and starch accumulation [37, 38]. When the activities of key enzymes related to sucrose metabolism were measured during different stages of cassava storage root development, it was also found that the cassava STs had significantly higher activities of invertase, sucrose synthase and hexokinase than both parents during the expansion period (Fig. 8). Studies have shown that during the rapid accumulation period of cassava starch, i.e., the expansion period, the main invertases involved in sucrose degradation in the storage roots are vacuolar invertase and neutral/alkaline invertase [39]. The differentially expressed invertase genes in our study were also vacuolar invertase genes and neutral/alkaline invertase genes, while the cell wall invertase gene in ST was not differentially expressed from its parents, indicating that this result was consistent with previous research. Therefore, the significantly higher starch content of the cassava ST relative to its parents during the expansion period is due to the joint regulation of three key enzyme genes that increase enzyme activities, resulting in a much higher degradation and utilization rate for sucrose in the storage roots and increased raw material for starch synthesis. The starch content of the cassava ST increased slowly during later stages of development and then nearly ceased to increase, indicating that the ST storage roots reached maturation in a short period of time, and their maturation time was greatly shortened relative to that of their parents. The activities of invertase, sucrose synthase and hexokinase in the cassava ST and its parents were measured during the mature period. It was found that the activities of invertase and hexokinase in the ST were significantly lower than those during the expansion period, which were significantly lower than those of the two parents, and the synthase activity in this period was similar to those in the expansion period and was not significantly different from those of the parents. The experimental results indicated that the cassava ST ceased to rapidly accumulate starch at this stage, and its starch content barely increased probably because the catabolic capacity of sucrose was limited by the activities of invertase and hexokinase, while sucrose synthetase was not a major regulatory factor. The enzyme genes related to starch synthesis were also not differentially expressed in ST and its parents, but further experiments are needed to verify whether these key enzymes are involved in posttranscriptional regulation. Therefore, we believe that the early filling of starch granules and the greatly shortened maturation period of the cassava ST storage roots are regulated by key enzyme genes involved in sucrose catabolism. These enzymes are active mainly during the formation period and early expansion period of the ST roots, making the first 8 months after planting the rapid starch accumulation period of the ST, and the starch content during this period far exceeds that in the two parents and does not differ from that during the mature period; thus, the ST storage roots can be harvested at this time. However, the rapid starch accumulation period of its parents is the later expansion and maturation stage, so their storage roots cannot be harvested until a 1 year after planting.

## Conclusions

In this study, we compared the physiological, agronomic performance and sucrose metabolism and starch accumulation from leaf “source” to storage root “sink” of ST and its parents at different growth stages, and combined their transcriptome data to elucidate the mechanism of ST precocious maturity. Compared with its parents, ST showed better agronomic characteristics, and the pattern of sucrose degradation and utilization in ST was different from that of its parents, which promoted the early maturity of its tuberous roots, and was mainly regulated by the activities of sucrase and hexokinase related to sucrose metabolism in tuberous roots. The results of this study can effectively determine the harvest period of high-yield and high-starch cassava, provide a theoretical basis for the screening of high-yield and early-maturing cassava varieties, and provide a basis for further genetic improvement of excellent cassava traits.

## Methods

### Plant materials

The experimental materials were asexual reproduction lines of a cassava ST (ST) and its parents, South China No. 5 (SC5♀) and South China No. 10 (SC10♂). All materials were planted in April at the Wenchang Experimental Base of Plant Resistance Genetic Engineering Group, Institute of Tropical Bioscience and Biotechnology, Chinese Academy of Tropical Agricultural Sciences, Hainan Province, China. Select mature, dense internodes, no damage to the stem surface and buds, and no pests and diseases in the middle stem section of 15-20 cm, and plant them in a flat way with the direction of the buds. The planting row spacing is 1 m × 1 m,, 30 of each varieties are planted. About 1 month after planting, replant ungerminated plants on a cloudy day. Cassava plants are relatively drought tolerant. The field management is based on the local climate in Wenchang, Hainan, China. Water is appropriate depending on the weather, and the soil can be moistened when it is dry.

### Determination of basic agronomic traits of ST and its parents during different stages of growth

The agronomic traits of the roots of 3 cassava varieties, SC5, SC10 and ST, in the field were measured every 35 d from approximately 95 days after planting and were measured in 5 periods, including the storage root formation period (95 days), expansion period (130 days, 165 days, 200 days) and mature period (235 days). Plants showing the same growth characteristics were selected for each period, and 3 plants of each variety were measured as biological replicates.

#### Blade-related indices

Leaf length, single decomposite leaf width and petiole length: The 4th complete leaf from the top was taken for measurement. Leaf area: Ten fully expanded functional leaves were randomly selected for leaf area measurements, the leaves were scanned by a DCP-7180DN scanner, and the leaf area was calculated using ImageJ software.

#### Cassava storage root-related indices

All tuberous roots of each plant were collected for the determination of storage root-related indicators. Determination of starch content requires slicing fresh storage roots of three representative cassava varieties and dried to a constant weight in an oven at 65 °C, ground to pass through a 100-mesh sieve and then dried and stored.

**Table:** 

measurement index	methods
Storage root length (cm)	the distance from the base to the tail of the root
Storage root diameter (cm)	the diameter of the thickest part of the root
Fresh weight (kg)	the fresh weight of an average single plant storage root
Dry matter rate (%)	the dry weight was obtained by drying at 60 °C to a constant weight dry matter rate (%) = dry weight/fresh weight
Total starch content (% fresh weight)	the 3,5-dinitro salicylic acid (DNS) method (Hsiao et al. [40])
The content of amylose (% dry weight)	using the plant amylose content Kit (micro method) of Suzhou Keming Biotechnology Co., Ltd.
The content of amylopectin (% dry weight)	total starch content-amylose content

### Measurement of photosynthetic characteristics of the ST and its parents’ functional leaves

During the mature period for SC5, SC10 and ST, three plants with the same growth potential from each variety were selected as biological replicates, and 10 fully expanded functional leaves from each plant were randomly selected for determination of leaf photosynthetic characteristics. The photosynthetic parameters of leaves were measured by using an LI-6400 Portable Photosynthesis System on sunny days with a 1200 μM/m2•s light source intensity, 500 μM/mol CO_2_ concentration and 30 °C leaf temperature.

### Chlorophyll content determination of the ST and its parent functional leaves

The leaf chlorophyll content was determined by picking the 4th fully expanded functional leaf from top to bottom during the mature periods of the three cassava varieties, SC5, SC10 and ST. The method was based on Hui et al. [41], with slight modifications. OD values were measured at 645 nm and 663 nm using a full-wavelength Enzyme-labeling Instrument SP Max-2300A, and the chlorophyll content was calculated according to the following formula:

$$\textrm{Chlorophyll}\ \textrm{a}\ \textrm{content}\ \left(\textrm{mg}/\textrm{g}\right)=\frac{\left(12.7 OD663-2.59 OD645\right)\times V}{1000\times W}$$

$$\textrm{Chlorophyll}\ \textrm{b}\ \textrm{content}\ \left(\textrm{mg}/\textrm{g}\right)=\frac{\left(22.9 OD645-4.67 OD663\right)\times V}{1000\times W}$$

$$\textrm{Total}\ \textrm{cholorophyll}\ \textrm{content}\ \left(\textrm{mg}/\textrm{g}\right)=\frac{\left(8.4 OD663+20.3 OD645\right)\times V}{1000\times W}$$

V is the amount of extraction solution mL; W is the weight of sample g.

### Soluble sugar content determination in functional leaves, stem phloem sap and storage roots of the ST and its parents during different growth periods

#### Sucrose, glucose and fructose extraction

Samples of cassava leaves/storage roots were taken at 95, 130, 165, 200, and 235 days after the three cassava products were planted to include the formation, expansion, and maturation stages of the cassava storage roots). For each sampling, 3 plants with the same growth from each variety were selected, the 5th and 6th fully expanded functional leaves were from the top of the stem downward, and the phloem and xylem in the middle of the storage root were taken and stored in an ultralow temperature refrigerator at − 80 °C. The extraction of sucrose, glucose and fructose from the cassava leaves and storage roots was performed according to the water extraction method of Yao [39]. The extraction of sucrose, glucose and fructose from the cassava stem phloem sap was performed according to the method of Pan et al. [5].

#### Sucrose, glucose and fructose content determination

Sucrose, glucose and fructose contents were determined in the samples by high-performance liquid chromatography with evaporative light-scattering detection (HPLC-ELSD). The chromatographic conditions were as follows: the column was a Waters x Bridge amino (Waters Amide 3.5 μm, 4.6 × 150 mm, USA); the mobile phase was acetonitrile:water = 70:30; the flow rate was 1 mL/min; the column temperature was 30 °C, and the drift tube temperature was 800 °C; the nitrogen flow rate was 2.0 L/min; the gain value was 2; the sample volume was 5 μL; the high-performance liquid chromatography instrument was an HPLC-1260 with an evaporative light scattering detector, ELSD-3300. The standard curves were prepared by accurately weighing the fructose, glucose and sucrose standards and preparing standard solutions of fructose, glucose and sucrose with the mobile phase at a concentration of 1 mg/mL. The sample volumes were 2, 5, 10, 15 and 20 μL. The standard products of all sugars were of chromatographic grade (Sigma, USA).

### Screening of metabolic pathways related to starch accumulation in ST storage roots and their key genes

The gene families of key enzymes for sucrose catabolism and starch synthesis in cassava storage roots were searched in the cassava genome database (https://phytozome-next.jgi.doe.gov/), which combines transcriptomic data to screen key metabolic pathways and differentially expressed genes associated with starch accumulation in cassava storage roots.

### Determination of key enzyme activities for sucrose and starch metabolism in the storage roots of the ST and its parents during different growth periods

The plant sucrose-related enzyme activity detection kit from Beijing Solarbio Technology Co., Ltd. was used to determine the sucrose invertase (INV), sucrose synthase (SuSy) and hexokinase (HXK) activities of the storage roots of the three cassava varieties during different growth periods according to the manufacturer’s instructions.

## Data Availability

The datasets supporting the conclusions of this article are available in the SRA database of NCBI repository (accession number: SRP151951).

## Abbreviations

SC5: *Manihot esculenta* Crantz South China No.5
SC10: *Manihot esculenta* Crantz South China No.10
ST: Cassava sexual tetraploid
UDPG: Uridine diphosphate glucose
Glu-6-P: Glucose-6-phosphate
Glu-1-P: Glucose-1-phosphate
ADP: adenosine diphosphate
AGPase: ADP glucose pyrophosphorylase
ADPG: ADP glucose
SSS: Soluble starch synthase
SBE: Starch branching enzyme
DBE: Debranching enzyme
GBSS: Granule-bound starch synthase
DNS: 3,5-dinitro salicylic acid
HPLC-ELSD: High performance liquid chromatography with evaporative light-scattering detection
INV: Sucrose invertase
SuSy: Sucrose synthase
HXK: Hexokinase
